# Towards Integrative and Multi-Scale Imaging with Recent Advances in Cryo-Electron Tomography

**DOI:** 10.3390/ijms27219519

**Published:** 2026-10-24

**Authors:** Simon Corroyer-Dulmont, Chloé Masson, Emmanuelle R. J. Quemin

**Affiliations:** 1 Okinawa Institute of Science and Technology (OIST), 1919-1 Tancha, Onna-son, Kunigami-gun, Okinawa 904-0495, Japan; 2 Institute for Integrative Biology of the Cell (I2BC), CEA, CNRS, Université Paris-Saclay, 91198 Gif-sur-Yvette, France

**Keywords:** high-resolution microscopy, cryo-electron tomography, focused ion beam milling, correlative light and electron microscopy, sub-tomogram averaging, near-native sample preparation, mesoscale context, cellular environment, multi-modal imaging, integrative structural biology

## Abstract

Cryo-electron tomography (cryo-ET) enables the three-dimensional visualization of molecular architectures within complex biological environments in a near-native state. In recent years, major advances in sample preparation, instrumentation, data acquisition, and computational processing have considerably expanded the scope of cryo-ET. Workflows that were once low-throughput and largely restricted to a small number of thin specimens and use cases can now be applied to many different samples from isolated macromolecular assemblies to cells, organoids, or tissues. In parallel, the advent of (i) automatic procedures for cryo-focused ion beam milling used for sample thinning as well as streamlined tilt series acquisition, (ii) improved methods for high-pressure-frozen specimens including lift-out, (iii) together with increasingly integrated reconstruction and processing pipelines are all contributing to improve throughput, reproducibility, and accessibility of the technique. Emerging deep learning tools further facilitate target identification, quality control with feedback loops, tomogram reconstruction, and downstream analysis by sub-tomogram averaging. Here, we discuss recent developments on different aspects of the cryo-ET workflow that are progressively bridging molecular and cellular scales in conjunction with multi-modal imaging. We also highlight current limitations and emerging technologies that may further extend cryo-ET capabilities, particularly in combination with complementary approaches toward integrative structural cell biology.

## 1. Introduction

Among the many imaging techniques used in Biology, electron microscopy (EM) has one of the highest resolving powers. While there are some specific applications of EM at room temperature (or conventional EM) and under cryogenic conditions (namely cryo-EM), the resolution capacity depends on the electron emission source used as it directly correlates with the properties of electrons making the beam, such as the wavelength and the acceleration voltage. Historically, Ernst Ruska won the Nobel Prize in Physics in 1986 for the development of the transmission electron microscope (TEM) [1]. About 30 years later, in 2017, the Nobel Prize in Chemistry was awarded to Jacques Dubochet, Joachim Frank, and Richard Henderson, for the development of cryo-EM “for the high-resolution structure determination of biomolecules in solution” [2]. On the hardware side, such advancement in the cryo-EM field from the formally known blob-ology era was made possible thanks to the increased stability of aberration-corrected scopes, the high quality of field emission guns, and the advent of direct electron detectors [3] used with energy filters to increase the signal-to-noise ratio under low-dose imaging conditions [4,5]. Nowadays, the continuous development of powerful software implementations also supports high-throughput data collection on modern microscopes and efficient processing afterwards with minimal user interventions.

Cryo-EM relies on vitrification and can be applied to a wide range of samples, which are considered in a frozen-hydrated state after cryo-fixation and formation of vitreous, non-crystalline ice [6,7]. In the absence of staining, chemical fixation or dehydration, structures are close to native, and densities relate directly to the biological content such as proteins, lipids, and nucleic acids [8]. Traditionally, this was implemented by plunge-freezing in liquid ethane or a mixture of ethane/propane cooled down to liquid nitrogen temperature after applying a thin layer of biomolecules in suspension on support grids [9]. After the so-called resolution revolution [4], the main modalities of cryo-EM include now microelectron diffraction of small crystals, single particle analysis (SPA) of isolated macromolecules, and tomography of pleomorphic and/or unique objects [10,11]. Recent applications encompass large protein complexes, flexible domains, and membrane proteins, to investigate conformational heterogeneity, compositional states, and structure–function relationships [12,13]. While sample preparation remains complex and specific workflows must be tailored on an individual case basis, the ultimate goal is to bridge the resolution gap between cellular and molecular biology towards integrative structural biology by increasing biological complexity and integrity [14].

Here, we discuss recent developments in the field that lead to a multi-scale continuum for cryo-electron tomography (cryo-ET), which has been classically applied either to cells or unique and pleomorphic objects in 3D. We focus mainly on various aspects of sample preparation, mentioning some specificities of data collection and processing, alongside making cryo-ET amenable to a broad range of specimens from macromolecular assemblies to multi-cellular organisms. Altogether, we touch on ongoing and future works that are likely to tackle key technical challenges beyond current correlative approaches and, thereby, move frontiers in structural cell biology towards understanding dynamic biological processes directly in situ as well as powerful integration with complementary techniques.

## 2. Main

ET relies on the principle of reconstructing a 3D volume in silico after the collection of 2D projection images in a TEM of a given region of interest (ROI) with typical tilt angle range between −60° to +60° and incremental steps of 2–3° [15,16]. The arrangement of such tilt-series is based on the data collection scheme applied, including continuous, bidirectional, or dose-symmetric, which contributes to the quality of data together with the field of view and resolution depending on the magnification used and pixel size selected [17].

### 2.1. Sample Preparation Aspects for Cryo-ET

#### 2.1.1. Cellular Cryo-ET on Vitreous Sections or Cryo Lamellae

**Figure 1:**
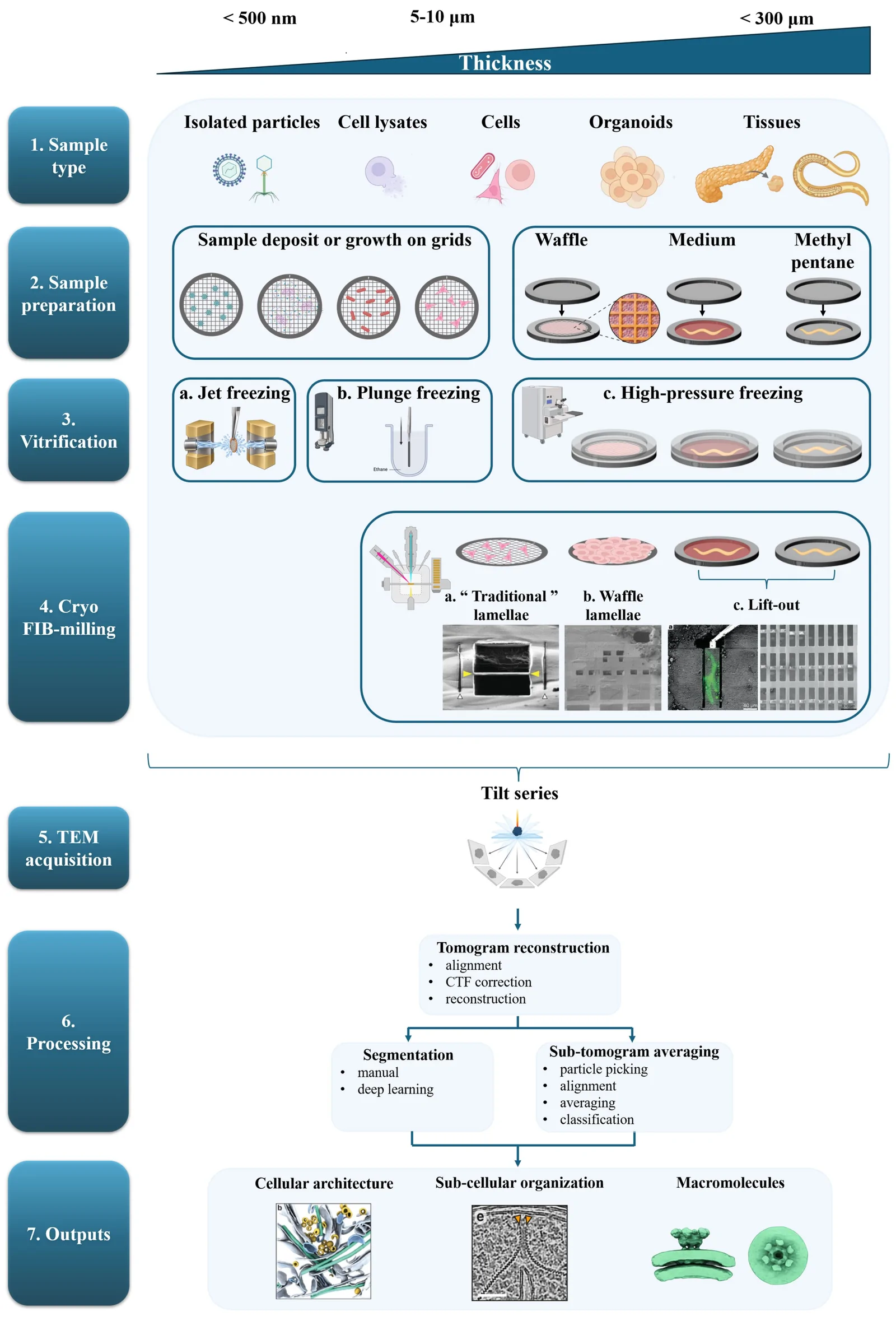
Cryo-electron tomography workflows from sample preparation to biological insights. Different vitrification methods are presented for sample preparation depending on the sample type (**1**–**3**). For thin samples, including isolated particles (<500 nm), cell lysates, and isolated cells (<10 µm), plunge-freezing is generally the most suitable method (**3.b**) [34]. This method necessitates a high concentration of particles and may cause stress to the cells during blotting. Furthermore, quality of vitrification may be heterogeneous for the same experiment and only assessable by cryo-focused ion beam (FIB), SEM, or TEM. Efforts have been made to automatize the process, to reduce the amount of samples needed, and to propose other vitrification methods such as jet freezing (**3.a**) [35,36,37,38,39]. High-pressure freezing is required for thick samples (>10 µm) such as monolayer of eukaryotic cells, organoids or tissue samples (**3.c**) [40]. The waffle method in particular (**2.** “Waffle”–**4.b**) is a specialized vitrification approach for cells or tissue that uses a high-pressure freezer instrument (see more detailed description in the text). Following vitrification, samples may undergo FIB milling (**4.a**,**b**) or lift-out preparation (**4.c**) before data acquisition of tilt series at the TEM (**5**). Primary data processing involves tilt series alignment, contrast transfer function (CTF) correction, and reconstruction of the 3D volume to generate the tomograms (**6.** “Tomogram reconstruction”). Subsequent analyses encompass segmentation (**6.** “Segmentation”) and sub-tomogram averaging (**6.** “Sub-tomogram averaging”) to enable visualization after rendering of cellular architecture, direct observation of sub-cellular organization, and medium to high-resolution structure determination of macromolecules in situ (**7**). Created with BioRender. Masson, C. (2026) https://BioRender.com/npgxj4r (accessed on 21 October 2026) and adapted for reproduction with permission from Klumpe et al., 2021 [41] (**4.a.** “Traditional lamellae”), Kelley et al., 2022 [42] (**4.b.** Waffle lamellae), Schiøtz et al., 2024 [43] (**4.c.** Lift-out), Nguyen et al., 2024 [44] (cellular architecture), Shimakawa et al., 2024 [45] (sub-cellular organization), and Zimmermann et al., 2023 [46] (macromolecules).

Initially, ET was applied to ultra-thin sections (typically below 100 nm in thickness) of biological samples embedded in resin after chemical fixation, dehydration, and staining with heavy metals [18]. First attempts of cryo-ET were performed on cell extracts or purified organelles [19,20,21], prokaryotic cells [22], pleomorphic viral particles [23], and flat or flattened eukaryotic cells [24,25] (<1 µm thick). Cryo-ET is primarily applied on such models, especially for rare, pleiomorphic, heterogeneous objects not suitable for SPA; however, it is also limited to the cell periphery if used standalone (Figure 1). Despite continuous improvement in sample preparation expanding SPA towards in situ analysis of physiologically relevant states with lysate-based or cell-opening approaches [12,26,27], integration of different systems is often required to recapitulate the full complexity of the cellular environment.

As a matter of fact, the specimens suitable for imaging are restricted in size by the mean free path of electrons making the beam. Therefore, sectioning using an ultramicrotome as traditionally employed in EM labs but under cryogenic conditions was proposed and coined cryo-electron microscopy of vitreous sections, CEMOVIS in short [28,29]. In practice, many technical challenges make CEMOVIS a highly demanding sample preparation step [30,31] and result in several drawbacks [32], such as attaching sections to grids efficiently or compression artefact [33], which limited its widespread adoption.

CEMOVIS has been successfully applied for cryo-ET [47,48,49,50,51] and is still considered as an alternative to the more commonly used method today: preparation of cryo lamellae using focused ion beam (FIB) milling [52]. FIB milling in cryo produces electron-transparent cross-sections, called lamellae (thickness typically between 150 and 300 nm), using the properties of the FIB to avoid mechanical stress introduced by mechanical sectioning [53]. Initially tailored for material science [54], this technique makes use of a dual beam scanning electron microscope (SEM) system and was later adapted for biological samples [55].

In general, for cryo-FIB milling, cells are deposited (e.g., bacteria or yeast cells) or even directly grown (e.g., mammalian cells) on support grids prior to vitrification via plunge freezing (Figure 1(3.b)). While the grids can either be coated and functionalized with poly-lysine or fibronectin to aid with seeding of adherent cells for example [56], more sophisticated positioning includes photo-micropatterning for attachment at predefined locations [57]. A cryo lamella consists of a thin section of the specimen that sort of supports itself on the grid by hanging on the surrounding bulk of the ice-embedded biological material after FIB milling (Figure 1(4.a)). Early on, some workflows used milling patterns to ablate material only at the top of the sample, resulting in a “wedge lamella”. However, difficulties in achieving the homogeneous and precise thickness desirable for TEM imaging and removing ice contamination from the bottom of the grid in particular led to a preference for milling from both sides [58,59]. This geometry of milling remains in use for standard workflows with better lamella polishing quality including a gas injection system (GIS) for sputter coating to protect from overmilling, to minimize curtaining, and to avoid charging of the sample during milling or imaging [60]. Indeed, sputtering is also applied as a final step to improve the conductivity of the surface of the lamella while the layer of platinum metal deposited is thin enough to not affect subsequent data acquisition for cryo-ET [61]. Additional improvements represent an active area of research spanning prevention of ice contamination [62,63], automation of the entire process [41,64,65], multi-step milling patterns, and stress relief “micro-expansion joints” [66], to reduce mechanical tension on the lamella [42,67,68,69].

In addition to commonly used FIB based on gallium as the ion source, there have been some recent developments of plasma-coupled FIB generated from nitrogen, xenon, oxygen, or argon [70]. They offer a higher milling rate and in some cases a reduction of milling-induced damage based on the properties of the ion source used, as well as the possibility of applying lower voltage [70,71,72]. Nowadays, many system configurations are commercially available with different hardware and functionalities from several manufacturers [73].

#### 2.1.2. Advancements of Cryo-ET Workflows for Organoids or Tissue Specimens

Apart from monolayers of some adherent cell lines and small bacteria or yeast in suspension (i.e., less than 10 µm-thick), plunge-freezing is not sufficient for proper vitrification of samples up to ~250 µm in thickness, and they require high-pressure freezing (HPF) instead [74,75] (Figure 1(3.c)). These thicker samples, such as organoids, tissues, or plants, are known to vitrify poorly, except if cryoprotectants are used [76,77,78], which may cause artefacts and even osmotic shock [79,80,81]. For very large specimens, sectioning with a standard vibratome setup, either embedded in soft agar [82,83] or not [44,84], can be done prior to HPF. Indeed, HPF is traditionally carried out in small aluminum carriers [74,75]. However, such devices are incompatible with subsequent cryo-ET data acquisition in TEM, and several solutions have been proposed over the years to circumvent this issue.

The first solution relies on modifying the carrier to make it compatible with FIB milling and subsequent cryo-ET imaging. This method offers high sample stability due to on-carrier milling and, in theory, minimizes direct contact during transfer. However, the current designs require cryo-ultramicrotomy using a specifically engineered holder [84,85,86]. Therefore, the amount of sample material to remove to obtain an observable lamella in TEM severely limits the throughput per carrier.

The second adaptation of the workflow involves placing a traditional EM grid inside the HPF carrier, which can then be directly used after freezing. This approach was initially demonstrated using *Caenorhabditis elegans* on EM grids placed in an HPF carrier filled with 2-methylpentane [78] (Figure 1(2)). Since 2-methylpentane has a low sublimation temperature (−160 °C), which is above the water devitrification temperature (−140 °C), the sample placed on the grid can easily be detached from the carrier, making it directly accessible for cryo-FIB milling at its surface [78,82,87]. However, the potential side effects of 2-methylpentane during the brief incubation period before freezing remain unclear. Additionally, its rapid evaporation at room temperature may lead to incomplete carrier filling, causing pressure-induced artefacts during freezing. Furthermore, the substantial amount of ice-embedded biological material that must be removed during milling limits, so far, the number of lamellae produced (e.g., up to 30 h per site using Ga^+^ FIB) [78]. Alternatively, the “waffle method” [88] (Figure 1(2–4.b)) is based on sample deposition on the back of the grid that is sandwiched between two flat carriers for HPF [89]. This imposes restrictions to the sample volume depending on the dimensions of the grid squares. By using different types of grids, this method can accommodate cell suspensions, organoids, and small tissues for cryo-ET [42]. However, there is still a large amount of material to be removed when compared to traditional cryo-FIB milling of cells grown or deposited on grids, thereby reducing throughput in terms of lamella production and potentially limiting the tilt range during cryo-ET acquisition [42,89].

Finally, another solution for performing cryo-ET on voluminous specimens after HPF is the “cryo-FIB-lift-out” method (Figure 1(4.c)), which transfers part of the sample from the carrier to a receiver EM grid using a micromanipulator and gripper [90]. Originally used in materials science [91,92], this method was later adapted for biological samples at room temperature in resin [93], but also under cryogenic conditions [94,95]. Since the demonstration of its feasibility nearly ten years ago [94], significant advancements are underway in terms of sample block transfer, attachment to the receiver, as well as many other innovative features to increase the throughput including the number of lamellae accessible from a single block [90].

Currently, typical lift-out procedure begins with rough milling around the ROI to make a block, sometimes also called a coarse lamella. The surface of the latter is then attached via material redeposition during FIB milling, while cold deposition of the platinum using the GIS remains another option. This connection to the sample can be done on an adaptor (e.g., copper or gold metal piece created from the receiver grid) attached to the needle of the micromanipulator [43,44] or directly to the needle tip [81]. Once the attachment is secured, the block is separated from the rest of the sample using FIB milling and subsequently transferred to the receiver grid. Furthermore, sections, approximately 2–4 µm in thickness, can be prepared iteratively from the block by FIB milling in the so-called “serial lift-out” or serialized workflows. In this case, the sections are attached to the receiver grid either at the grid bar or on the grid mesh [43,44].

In brief, the first attachment method, i.e., to the grid bar, provides higher stability, especially with dual-site attachment using FIB milling redeposition. However, it restricts the block and section size to the grid mesh dimensions and geometries. On the other hand, attachment to the grid mesh offers more flexibility in terms of sample size and placement. In this case though, section separation and attachment rely on electrostatic interactions, which can sometimes be insufficient and lead to section loss and small output. Once attached, sections can be automatically polished into lamellae with similar workflow and the software used for cell milling (see above). Despite recent improvements, the cryo-FIB-lift-out approach remains technically challenging with low-throughput and, more critically, lacks automation for the vast majority of the steps.

Regardless of the method used, cryo-ET on tissue faces several challenges besides being an active area of research [44,81,96,97]. One difficulty mentioned already is the amount of material that must be removed to obtain a thin lamella transparent for imaging in TEM. This results in a lower lamella throughput for tissue samples compared to cells, due to a long milling time. The recent development of plasma FIB milling at cryogenic temperatures provides a solution to mitigate this. With an increased ablation rate for cryo-FIB milling, the preparation of lamella using a xenon-based ion beam reduces the time to approximately 50 min per site for samples frozen using the waffle as the sample preparation method. Additionally, plasma FIB systems appear to limit milling-induced damage from a depth of 60 nm in the case of gallium ions [71,98] to 30 nm with xenon, affecting surfaces on both sides. Such damage can be further minimized with low voltage [72], such as 8 kV instead of 30 kV used for gallium ions. However, low-voltage milling leads to an increase in probe size, and mitigating the associated curtaining side-effect on the lamella would require the development of new milling protocols or practical adaptations of scopes tailored for biological applications.

Another challenge in cryo-ET is the lack of distinction between the ROI and the rest of the sample during imaging, which is proportionally amplified when working with tissue specimens. Indeed, this makes targeting the ROI not only difficult during cryo-FIB milling but also its identification unreliable in datasets after cryo-ET without complementary or orthogonal approaches for validation and interpretation of the phenotypes observed. Of note, the vast majority of the sample volume is ablated and thus lost during the lamella preparation process due to cryo-FIB milling. Combined, these inherent features mean that the lamella represents only a tiny snapshot of highly dynamic molecular events occurring within the crowded and much larger volume of the cell.

### 2.2. Better Together: Cryo-ET Along with Correlative and Integrative Frameworks

#### 2.2.1. Correlative Light and Electron Microscopy or CLEM

**Figure 2:**
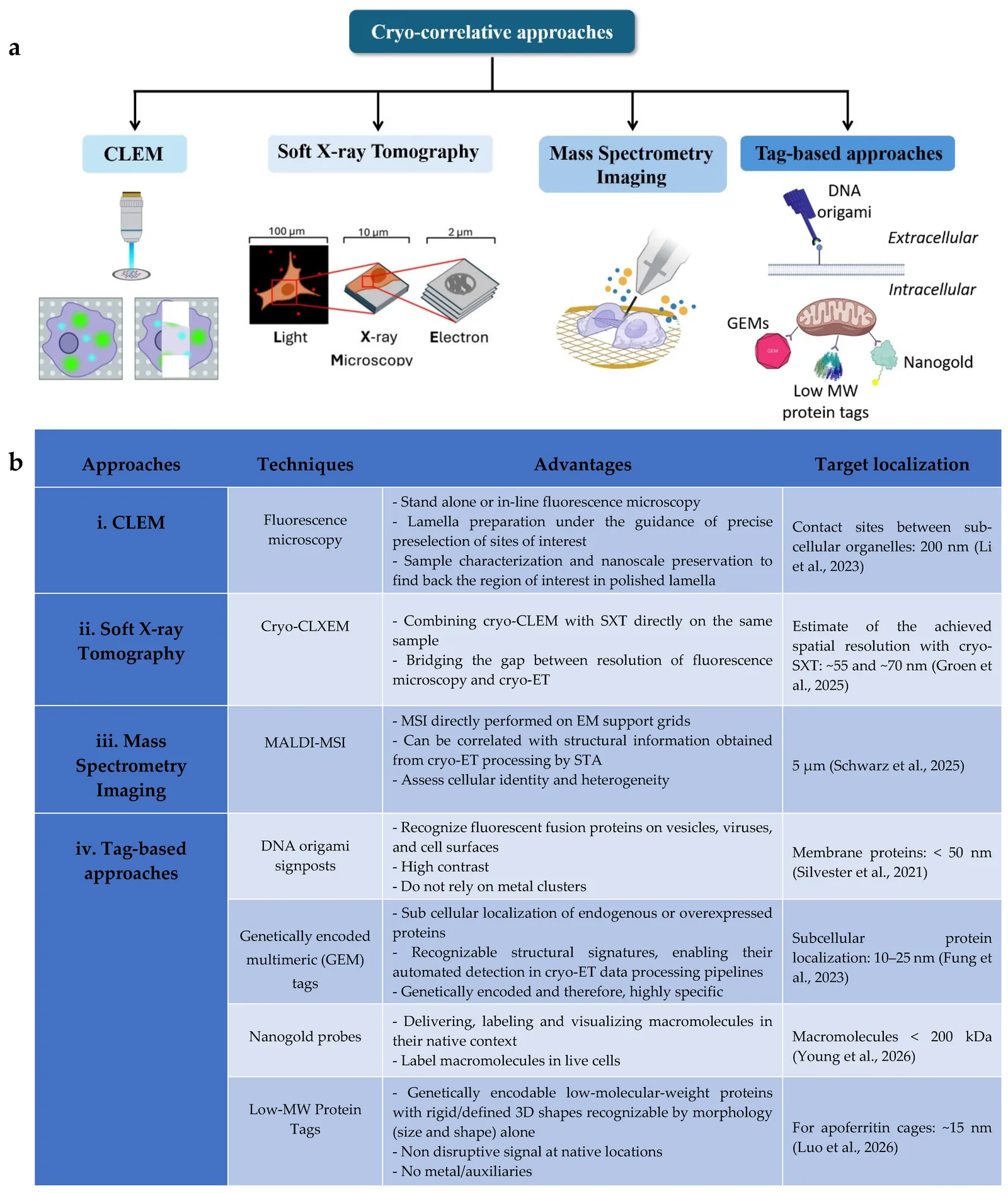
Cryo-correlative approaches used in cryo-electron tomography as complementary or integrative workflows. (**a**) Overview of four major approaches used in conjunction with cryo-electron tomography (cryo-ET): correlative light and electron microscopy (CLEM), soft X-ray tomography (SXT), mass spectrometry imaging (MSI), and tag-based approaches. (**b**) For each method, representative techniques with main advantages and the achievable target localization are given in a table. (**i**) CLEM integrates fluorescence microscopy with cryo-ET to guide target identification and lamellae preparation. (**ii**) SXT bridges the resolution gap between light and electron microscopy, while (**iii**) MSI provides detailed molecular information that can be correlated with structural data obtained by cryo-ET. (**iv**) Many different tag-based approaches can facilitate the localization of specific proteins and macromolecular complexes that they form at the cell surface or directly on the inside. Representative and seminal publications are provided for each approach: Li et al., 2023 [103], Groen et al., 2025 [104], Schwarz et al., 2025 [105], Silvester et al., 2021 [106], Fung et al., 2023 [107], Young et al., 2026 [108], Luo et al., 2026 [109]. Created with BioRender. Masson, C. (2026) https://BioRender.com/87evxjf (accessed on 4 September 2026) and adapted from Groen et al., 2025 [104] for SXT panel with permission.

Over the years, cryo-ET has been combined with many other techniques such as light microscopy (LM) for extensively used correlative pipelines, either at room temperature or under cryogenic conditions after vitrification. In practice, the strengths and weaknesses of cryo-ET and LM make them genuinely complementary [99] (Figure 2a,b(i)). As an example, LM can be applied to mitigate the low temporal resolution and difficulty in identification of ROI by cryo-ET alone [100,101]. Indeed, LM can become handy at different stages of the whole cryo-ET workflows described above for various purposes: (i) before vitrification, room-temperature LM allows temporal analysis of the event(s) or phenotype(s) of interest; (ii) after vitrification, cryo-LM can aid with localization of the ROI(s) in the FIB-SEM prior to and even after cryo-FIB milling [102].

While targeting the ROI in the xy plane is relatively straightforward, correlation in the z dimension suffers from lower resolution in this direction. To mitigate this, several use cases have been described for accurate ROI targeting during lamella milling. The most straightforward option relies on an integrated fluorescence light microscope (iFLM) inside the FIB-SEM chamber to monitor the fluorescent signal, either prior or during preparation of the lamella [76,110,111]. Although automation capabilities are being implemented, the switch and registration between the different imaging modalities is primarily done manually given that the objectives of iFLM in most commercially available FIB-SEMs are not positioned at the coincidence point compared to the electron and ion beams.

In microscopes with such specific hardware configuration, recent developments have enabled fluorescence monitoring in real-time during milling [112] or in-between the lamella preparation process. However, one remaining issue is that after the final polishing step, most of the fluorescent signal from the small particles becomes undetectable, creating a gap between observations by LM before polishing and the final lamella analyzed by cryo-ET afterwards [113,114].

In parallel, fluorescent beads that are easily identifiable in the FIB-SEM, either with iFLM, the electron beam, or the ion beam, can be used for correlation purposes. Such an approach theoretically allows precise lamella placement before automated milling. However, grid and sample movement because of distortion or drift during milling, in particular, can lead to misalignment and inaccurate targeting of the lamella. Additionally, this requires fluorescent beads to be distributed homogeneously and near the ROI, which is incompatible with large samples such as tissue or organoids.

Along this line, approaches based on molecular tags for target identification have continuously evolved and many different strategies tailored to cryo-ET workflows are now available, including metallic nanoparticles such as gold, metal-chelating proteins, and nanostructures based on nucleic-acids or protein assemblies (Figure 2a,b(iv)). These represent promising areas for development, which have been reviewed at length recently [115] and are not described in more detail here.

#### 2.2.2. Fill the Gap Between LM and Cryo-ET Using Whole Cell Volume Imaging

While cryo-CLEM is the most common approach used to target ROIs within biological samples, it is limited by the resolution gap between these two imaging modalities. The lower resolving power of LM, combined with poor spatial accuracy in localizing fluorescent signals within samples, limits targeting prior to or during milling. To overcome this, two other approaches have been implemented: the cryo serial sectioning for volume imaging and cryo soft X ray tomography (cryo-SXT).

##### Combining Cryo Serial Sectioning for Volume Imaging with Cryo-ET

Cryo serial sectioning or so-called slice and view is a volume EM method that provides an isotropic 3D representation of a sample and its ultrastructure within a large spatial context. In practice, it consists of using the FIB to ablate a thin layer of material from the sample surface and then to come to the SEM for imaging (secondary or backscattered electrons with specific detectors) in a stepwise and repetitive manner [116,117].

While such a volume EM method has been widely used for chemically-fixed and resin-embedded samples at room temperature, its application to cryo-samples remains more limited due to several drawbacks such as charging effects [118,119] or curtaining artefacts [120]. Although GIS application can reduce topographic variations at the surface, curtaining remains an important impediment to high-quality imaging, due mainly to non-uniform sample surfaces caused by ice contamination, crevasses, or cellular heterogeneity. Additional issues including beam-induced damage, ice contamination, microscope instability, long acquisition times, and low image contrast further increase the challenges associated with volume EM under cryo conditions. However, cryo slice and view FIB-SEM permit contextualization of part of the ROI surrounding environment and assist target localization when applied to the region next to the actual lift out site, for example. Lamella preparation can also be guided by SEM imaging, with progressive milling of the top surface until the ROI becomes visible, followed by milling of the bottom surface to reach a thickness suitable for cryo-ET [121]. Of note, progress in many different areas has been reported including the implementation of new ion beam sources [119,120], optimized scanning strategies [118,122,123], and automation [41,124].

##### Combining Cryo-SXT and Cryo-ET

Cryo-SXT is based on low-energy X-rays to image frozen-hydrated samples, typically an entire intact mammalian cell in 3D at nanoscale, without the need for prior chemical fixation, staining, or sectioning [125,126,127]. This is enabled by imaging in the “water window”, an energy range in which oxygen and carbon-containing structures absorb incoming photons at different rates, producing natural inherent contrast for biological samples, in particular. Within this energy range, soft X-rays can penetrate up to 10 µm in depth, allowing imaging of most cell types as a whole. Although having less spatial resolution than TEM (~30 nm isotropic resolution for cryo-SXT), this technique has emerged as a powerful tool to visualize 3D cellular volumes at higher lateral and axial resolution compared to LM with little effect of radiation damage [128]. Consequently, it has also been combined with other imaging modalities, including cryo-correlative light and X-ray microscopy (cryo-CLXM) [129,130,131] and multiscale experiments involving cryo-EM/ET (cryo-CXEM) [104], with a wide range of studies already reported (Figure 2a,b(ii)).

In the context of cryo-CXEM, cryo-SXT can be used in combination with techniques such as cryo-ET, cryo-FIB-SEM, and cryo-lift-out, providing a holistic workflow empowered by accurate correlation between multiple imaging techniques and across different length scales to probe cells and macromolecules at the subcellular and molecular level. Indeed, most recent integrative workflows have been implemented combining cryo-LM, cryo-SXT, cryo-FIB milling, and cryo-ET directly on the same sample, providing a comprehensive multimodal imaging study under native conditions for investigating complex samples and small specimens (nanoparticles), as well as rare cellular events. Although previously limited to a few large synchrotron facilities worldwide, the development of “benchtop” compact soft-X ray devices [129,132] with plasma sources [133] is more than likely to improve the accessibility to many more laboratories in the coming decades and enhance research applications in the fields of structural cell biology and medical biology, in particular.

#### 2.2.3. Integrative Approaches Towards Structural Cell Biology In Situ

While several approaches, such as CLEM, tag-based methods, and direct inference from cryo-ET data, enable cell identification, these can be rather time-consuming and/or tedious. Moreover, these approaches have limited ability to report on chemical identity or molecular heterogeneity of the samples in a comprehensive systematic manner and at the spatial scales often required to inform cryo-ET analysis. The emerging field of structural and visual proteomics relies on an ensemble of techniques partly based also on mass spectrometry (MS) to characterize cellular states and composition in terms of proteins (proteome), protein networks (interactome), or lipids (lipidome).

Given that MS and cryo-EM/ET provide highly complementary information on samples even when they are performed in parallel, combining them has been instrumental for the advancement of integrative structural biology [134,135]. The many MS techniques now available are indeed label-free and cover metabolic, structural, as well as functional investigation of biomolecules [136]. Proteins, lipids, nucleic acids, and the biomolecular assemblies they form can be investigated (i) either in isolation, in bulk or directly in biological systems and also (ii) under a wide range of denaturing conditions (such as peptide fragmentation and addition of chemical cross-linking agents) or by native MS [137]. Hence, MS-based technologies have greatly contributed not only to the field of biochemistry but also to our current understanding of cellular, molecular, and structural biology. The nice correlation between pipelines for cryo-ET and biomolecular MS has been recently exemplified with integrative data processing for cross-linking MS, in particular when applied to single *Mycoplasma pneumoniae* [138] as well as bigger mammalian sperm cells [139].

Mass spectrometry imaging (MSI) represents another well-established approach for investigating the chemical identification and localization of biomolecules within intact samples at low to medium range resolution, thereby especially complementing structural imaging by cryo-ET (Figure 2a,b(iii)). Among MSI methods, matrix-assisted laser desorption/ionization (or MALDI in short) MSI is unbiased and powerful in identifying the spatial distribution of a vast amount of small metabolites, lipids, and proteins within sections of tissues, individual cells, or even at the subcellular level, which generates highly detailed molecular maps focusing mostly on cellular heterogeneity and metabolic state [140,141]. A combination of cryo-ET and MALDI-MSI was recently applied to the same specimen, aiming to directly assess cellular identity on grids following data collection at the TEM [105]. In brief, this innovative work encompasses a standard cryo-ET pipeline from cell growth on EM support grids to cryo-ET data collection and processing; followed by freeze-drying, MSI acquisition, and specific data analysis. Although technically challenging, this combined approach could infer intercellular heterogeneity, detect different species or multiple cell types in mixed populations, and even provide insights into subcellular organization. Notably, a dedicated software environment has been developed to integrate all modalities of this pipeline [105]. One of the latest publications on the matter also describes chemical imaging using secondary ion mass spectrometry (or SIMS in short) carried out with cryo-FIB-SEM, which is compatible with cryo-LM, and coupled with cryo-EM analysis of bacteria as well as cryo-ET data collection on lamellae of eukaryotic cells [142]. These represent proof-of-principle experiments towards integration with already established cryo-CLEM workflows. Despite their clear potential, particularly as they avoid the need for genetic modification, the use of exogenous probes, or any other type of potentially significant cellular perturbation, these approaches remain quite niche and low throughput at the moment, as they require specialized instrumentation.

For sequential studies, the output of complex pipelines encompassing direct identification and sample characterization by MS, subsequent to cryo-ET sample preparation and data collection, is nevertheless striking and holds great promise for broader applications in the near future. Notwithstanding the results from MALDI-MSI, it does appear to be rather limited in terms of spatial resolution (5 µm pixel size [105]) when compared to SIMS data that are in the sub-micrometer resolution regime. However, SIMS is generally tailored to small or inorganic molecules, leaving biomolecules such as lipids or proteins undetected. Therefore, significant challenges remain, and the results of ongoing research will contribute to the development of more user-friendly and automated MS-based methods readily compatible with cryo-ET standard workflows to analyze the interplay of dynamic biological systems in situ at multiple scales.

### 2.3. Cryo-ET Data Collection and Processing

#### 2.3.1. Acquisition Schemes

In practice for cryo-ET, frozen-hydrated samples are physically tilted inside the column of a TEM (Figure 1(5)) to generate a series of 2D projection images focusing on a given ROI at each predefined tilt angle. Subsequently, those so-called tilt series are computationally aligned as stacks and reconstructed in silico into tomograms representing the sample volume in 3D in near-native state [143].

Depending on the stage geometry, individual images of the tilt series are usually collected from an angle range between −60° and +60° with an increment of 2 or 3°. In practice, stage mechanics impose that tilting is restricted to ±70°, plus there is also increased sample thickness at high tilt, which further limits the signal to noise ratio due to multiple electron scattering events. Overall, such tilting schemes lead to non-homogenous sampling of information in 3D referred to as the “missing wedge” of information in Fourier space and causing deformation artefacts in real space. Hence, tomograms display anisotropic resolution with elongation of objects in the z dimension and a loss of information for features found perpendicular to the tilt axis.

Moreover, frozen-hydrated samples observed under cryogenic conditions are highly sensitive to the electron beam and therefore must be imaged using low dose settings while each acquisition contributes to the cumulative electron dose applied to the sample [144]. As a result, images acquired at later points in the series are collected after accumulation of beam-induced radiation damages, leading to sample distortion, bending or drift, in addition to reduced high-resolution information along with elevated noise and artefacts [145].

Classical tilt schemes consist of either unidirectional or bidirectional data collection with variations in the increment angular range (e.g., linear interval, variable, or proportional to cosine). Nowadays, however, common cryo-ET practice relies more often than not on dose-symmetric data collection introduced in the 2010s [17]. In the unidirectional or continuous tilt scheme, acquisition starts from one of the highest tilt angles and proceeds continuously in one direction, which is particularly utilized for resin-embedded samples prone to resin shrinkage during illumination that can be more easily accounted for during alignment. In the bidirectional tilt scheme, acquisition begins at 0°, and the two tilt branches are acquired sequentially as separate segments towards positive and negative high angles. This approach was particularly suited for automated data acquisition; however, in this case, there is the “jump-start” issue because of unequal distribution of cumulative radiation damage across the two branches. In the dose-symmetric tilt scheme, acquisition starts at 0° and proceeds symmetrically in the positive and negative branches in parallel (e.g., 0°, −3°, +3°, −6°, +6°, … up to ±60°). This approach preserves the high-resolution information at the lowest tilt angles with gradual electron dose accumulation, facilitating processing for stack alignment and sub-tomogram averaging (STA) [17].

A phase plate is a small device placed in the back-focal plane of the objective lens inside the column of the TEM and used for electron modulation with phase shifting [146]. It has regained attention in expanding the capabilities of both cryo-EM and cryo-ET by increasing contrast and thus aiding downstream data processing [147]. Although practical limitations have hampered the potential widespread applications, improvements in novel designs and prototypes are seamlessly being reported [148,149,150]. Indeed, biological samples are considered weak-phase objects composed of light elements and generate little intrinsic contrast under TEM. To mitigate this, the performance of the Volta Phase Plate has been particularly powerful for a number of use cases, including for cryo-ET studies [147,151].

Finally, the dual axis tilt scheme has also been under the spotlight recently where the grid undergoes 90° stage tilt thanks to rotational holders. The goal is to obtain additional data on a given ROI after computing, but collected on two different orthogonal axes compared to traditional single axis tomography, which increases the wealth of information sampled in 3D and minimizes the effect of the missing wedge [67,152]. Given that samples under cryo conditions are highly sensitive to radiation damage, the limited low dose budget has to be taken into account and distributed accordingly across the two tilt series to be collected on the same spot, which has an impact on tomogram reconstruction and downstream processing aspects. Importantly, combined with phase plate-induced contrast enhancement, dual axis cryo-ET provided new insights into the structure of viral particles and the interplay with cellular components of the cytoskeleton for Ebola virus, in particular [153].

#### 2.3.2. Software Implementation for Automatic, Fast and Reliable Data Collection

Several software implementations are available for automated data collection in TEM encompassing some with specific configurations for cryo-ET: EM-Tomo (TVIPS GmbH), Leginon [154], SerialEM [155,156], TOM [157], Tomography (Thermo Fisher), and USCF Tomography [158].

Notably, SerialEM is one main academic software package, which has been predominantly used by the cryo-ET community [155,159]. Indeed, it is freely distributed as open-source and offers a versatile platform with a great degree of flexibility. The user has full control over the microscope settings to carry out customized acquisition pipelines, albeit at the cost of a steeper learning curve to find your way around the interface and get to know the many control panels and various functionalities available.

Compared with other cryo-EM imaging modalities such as SPA or microcrystal electron diffraction, cryo-ET remains relatively slow and challenging in achieving high resolution structure determination by STA down the line (Figure 1(6)). Once ROIs have been identified (usually by eye in a manual way but progress is being made for more automated targeting approaches), traditional protocols for tilt series acquisition require one individual tracking position in close vicinity and positioned along the tilt axis for the acquisition of each tilt series at the target(s) selected. Even though tracking and focusing done at each tilt can be on the same spot, the number of suitable sites for cryo-ET are hence restricted not only by their (random) distribution on the grid but also by the efficiency of high-quality imaging per se depending on the stage geometry, microscope optics, and detector setup. Innovative data collection strategies for cryo-ET are moving towards parallel and even continuous schemes to increase speed and output [160].

Apart from the methods for predictive correction modeling of shifts applied either as precalibrations [161] or monitored in real time, the concept of fast “continuous” data collection is still in its infancy [162,163,164]. Conversely, multishot tomography based on beam image shift, or BIS in short, has been proposed as another high-throughput strategy spanning BISECT (for BIS to speed up Electron Cryo-Tomography) [165] and on-axis multishot scheme [166]. Notably, the latest implementations for parallel data collection called PACE-tomo [167] and SPACE-tomo [168] available as scripts in the SerialEM interface have been readily adopted in many laboratories and core facilities. Other recent work developed continuous recording during beam image shift (CR-BIS) to optimize camera time allocation, thereby accelerating data acquisition for both cryo-ET and SPA [169]. These newly implemented pipelines are not only tailored for fast acquisition to improve current cryo-ET capabilities even on high end scopes but are also compatible with advanced processing by STA at high resolution.

Therefore, there has been a steady development of strategies that are better suited for lamellae prepared by cryo-FIB milling. These often contain dense biological information of high interest but are confined to a limited area. Tilt series are typically acquired with sufficient spacing between targets to avoid dose accumulation and the cumulative effects of radiation damage. Consequently, neighboring tomograms often lack continuity, making it difficult to reconstruct the overall architecture of a specimen or to grasp the mesoscale context in the crowded cell interior, for example. When contextual information is required, intermediate-magnification tomograms (~6000×) can be acquired to image an entire lamella or large ROI within a few minutes and at very low dose [170]. Alternatively, montage tomography can be used to collect multiple overlapping high-resolution tilt series ultimately covering a vast field of view [76,171,172,173]. However, issues arise with montage setups, and specimens need to tolerate the additional electron dose associated with image overlap used for stitching. Furthermore, tile stitching and tilt series blending for montage reconstruction remain technically challenging and are not yet integrated into the most commonly used processing workflows (see Data Processing in the section below).

In the mid- to long-term, the establishment of multi-grid workflows similar to SPA is in reach. Automation of grid mapping, identification of ROI (e.g., lamellae), selection of targets for collection of tilt series, on-the-fly monitoring, and remote reporting in a multi-grid framework will be highly beneficial in a number of ways. First, it will make efficient use of state-of-the-art microscope time in a reliable manner and with minimal user input. Second, it will ensure the highest quality datasets with relevant phenotypes, events, or features under investigation for integrative structural cell biology. Furthermore, the combination of cryo-ET together with SPA acquisition strategies such as GisSPA [174] offer promising directions for high resolution structure determination in situ [12,26]. Overall, this could streamline data collection parameters to populate public repositories and provide ample room for many more exciting scientific discoveries.

#### 2.3.3. Tomogram Reconstruction and Other Strategies for Data Processing

Data processing in cryo-ET is a complex and rapidly evolving field with new methods and often lab-specific software packages being introduced at a high pace without integration within a cross-platform grand scheme. Unlike SPA for which CryoSPARC [175,176] and RELION [177,178,179] represent widely adopted, standardized, and relatively user-friendly workflows, cryo-ET still lacks a universally accepted processing pipeline and clear guidelines for best practice. Driven by the community, ongoing promising developments such as Warp [180] with M [181] and in RELION [182] or also recently, the bran new platform Relay, https://github.com/warpem/relay/tree/main (accessed on 21 October 2026) are being implemented. This lack of standardization and clarity with more streamlined workflow(s) remains a significant barrier for newcomers and limits the broader adoption of STA approaches for structure–function analysis of macromolecules in situ.

A comprehensive discussion of cryo-ET data processing to grasp the ins and outs would warrant a dedicated review by itself. Therefore, in the following section, we provide a brief summary, hinting at recent methodological developments and future perspectives. Readers interested in detailed descriptions of cryo-ET processing methods are referred to the reviews cited below [183,184].

Initially, tomogram reconstruction typically consists of several successive steps: movie frame alignment, tilt series alignment through global and local refinement, CTF estimation and correction, dose weighting, and reconstruction by back-projection or other iterative methods (Figure 1(6)). One of the most widely used software suites for tomographic reconstruction is the IMOD package, which spans a wide range of applications including montage alignment, tomography, CTF correction, filtering, visualization, and segmentation [185,186].

Historically, precise tilt series alignment relied on fiducial markers, typically gold beads added to the specimen prior to vitrification. Tracking of these fiducials was done throughout the tilt series and used to determine the alignment parameters required for high-quality tomogram reconstruction. Here, automated batch processing was possible to a certain extent but relatively slow and often limited to a single or similar datasets.

While fiducial-based approaches remain highly effective, they are generally not applicable to lamellae because of a lack of fiducials. In such samples, alignment of the tilt series instead relies on patch tracking, in which features contained in image areas or sectors distributed across the field of view are tracked between tilts. Because patch tracking depends exclusively on the inherent content of the specimen, its accuracy can be limited, particularly in low-contrast or heterogeneous cases. The introduction of projection-matching approaches, first with Protomo [187] and later with AreTomo [188], significantly improved marker-free alignment procedures. These methods iteratively compare experimental projections with reprojections of an intermediate reconstructed volume, enabling highly accurate alignment approaching the quality obtained with gold fiducials. In addition, AreTomo2 introduced efficient batch processing capabilities that allow rapid reconstruction of large datasets through a relatively simple command-line interface. More recently, AreTomo3 embedded in AreTomoLive has expanded this framework by integrating additional preprocessing steps, including motion correction, denoising, dose weighting, and CTF correction, within a unified reconstruction workflow [189].

Recent developments have explored the use of deep learning approaches also for robust, automatic, and unsupervised tomogram reconstruction with MissAlignment [190], which employs convolutional neural networks to optimize alignment parameters for tomographic sub-volumes based on a “quality” score. The method additionally evaluates whether regions containing little structural information can contribute less to the optimization process and thereby reduces the risk of artefacts in heterogeneous specimens. Initial results indicate that structures obtained by STA from tomograms reconstructed using this new approach can reach resolutions comparable to those achieved with ultimate reference-based alignment methods. However, computational costs remain substantial even with processing done in batches of sub-volumes and total times, currently on the order of approximately one hour per tilt series depending on the computational infrastructure.

The increasing automation in tomogram reconstruction has made it possible to process large datasets with minimal user intervention and on-the-fly [189,191,192]. In practice, however, many tilt series contain low-quality images resulting from contamination, specimen drift, charging, or the appearance of lamella edges within the field of view. These defective tilts can significantly degrade the quality of the final reconstruction [193]. A common strategy consists of excluding low-contrast tilts, which is often sufficient to remove images affected by lamella-edge artefacts but is less effective for other sources of image degradation. More recently, deep learning approaches have been proposed to automatically identify and exclude poor-quality tilts prior to reconstruction [194]. Such methods are likely to contribute to the implementation of fully automated quality control in cryo-ET processing pipelines.

Downstream processing steps encompass contrast enhancement for visualization, target localization, sample context characterization, and eventually quantitative analysis (Figure 1(6)). The possibility of restoration methods from the advent of deep learning algorithms has to be treated with careful consideration on an individual case basis with appropriate control measures. However, these are useful in facilitating other aspects of the workflow and paving the way for the full integration of the many tools now available. They encompass either visualization aids [195,196,197,198,199,200] as well as quantitative methods for particle picking [201,202,203,204], denoising and missing wedge compensation [205], image segmentation and pattern recognition [206,207,208], or characterization of macromolecular heterogeneity [209,210]. In parallel, automatic reconstruction and on-the-fly feedback loops offer entirely new avenues for research using cryo-ET [194]. These further represent a prerequisite to make the most out of existing STA pipelines for high resolution structure determination of macromolecules directly in a physiologically relevant context [181,182,211,212].

## 3. Conclusions and Perspectives

Since its development, cryo-ET has proven to be a powerful technique for visualizing the molecular environment of a wide variety of biological samples in their near-native state and at medium to high resolution [213]. The past decade has seen numerous software and hardware developments enabling increasingly automated workflows and progressively shifting the bottleneck from obtaining suitable samples for cryo-ET towards data mining with advanced processing pipelines. Key advancements include the automation of cryo-FIB milling, enabling high-throughput lamella production from cells with improved reproducibility [41,64,65], automated acquisition across large areas of lamellae using deep learning-assisted target recognition [168], and automated tomogram reconstruction [189,192]. Despite substantial improvements, the application of cryo-ET to tissue samples through lift-out techniques remains challenging, slow, and relatively low in terms of throughput [214]. However, the development of plasma-based FIB for faster milling [70], together with recent advances in automated lift-out procedures that can also be assisted by deep learning [65], offers promising prospects for making this technique more broadly accessible and overall expanding the possible output from cryo-ET.

Artificial intelligence (AI)-based tools are currently transforming several aspects of the field (see review [215]). First, AI-assisted feature recognition is facilitating the automation of sample preparation, as illustrated by examples presented hereinabove. Second, it can accelerate and improve data acquisition through automated, fast, and reliable targeting of ROI as well as efficient analysis of many images with low signal to noise ratio [196,197,216,217,218]. Finally, data processing is also evolving rapidly with new approaches developed for segmentation [219], particle picking [219], and atomic model building. In the latter area, AlphaFold and related structure-prediction methods [220,221,222] have had a particularly profound impact by facilitating the interpretation and modelling of cryo-EM density maps and will continue to do so for cryo-ET in the near future (see review [223]).

In addition, several hardware improvements offer exciting opportunities. First, cryo-scanning transmission electron microscopy (cryo-STEM) could facilitate the investigation of specimens thicker than conventionally done for cryo-ET with thin lamellae, including samples exceeding 500 nm in thickness [224]. Second, correlative approaches combining cryo-SXT with cryo-ET could provide an alternative to the destructive volume EM slice-and-view approaches based on cryo-FIB-SEM, while providing whole-cell contextual information at the nanometer scale [104,129,133]. Finally, the development of laser phase plates could substantially improve image contrast at low defocus values, potentially facilitating the visualization and identification of biological structures in the crowded cellular content while preserving high-resolution information [225].

Overall, the different aspects of cryo-ET presented in this review highlight a promising future for the field. Continued developments are more than likely to enable the investigation of increasingly complex and physiologically relevant specimens, including tissues and organoids combining SPA and cryo-ET [26]. Furthermore, new user-friendly and integrated software packages are progressively alleviating one of the major challenges in cryo-ET data processing for newcomers and non-specialists. Together, these developments are expected to broaden the range of biological systems accessible to cryo-ET while increasing both the number and the resolution of structures that can be obtained by STA for dynamic macromolecules as well as their interplay in the cellular context and under near-native conditions.

## Data Availability

No new data were created or analyzed in this study. Data sharing is not applicable to this article.
